# A qualitative study of the factors that help the coping of infertile women

**DOI:** 10.1002/nop2.1062

**Published:** 2021-09-28

**Authors:** Suvi‐Tuuli Halkola, Meeri Koivula, Anna Liisa Aho

**Affiliations:** ^1^ Faculty of Social Sciences Health Sciences Nursing Science Tampere University Tampere Finland

**Keywords:** coping, infertility, qualitative research, women

## Abstract

**Aim:**

The study describes the factors that help the coping of infertile women.

**Design:**

A qualitative study.

**Methods:**

Purposive sampling was used in gathering the data from the Finnish Infertility Associations closed discussion forum on Facebook. An electronic questionnaire included structured background questions and a qualitative open‐ended question related to the factors that help coping. In total, 101 women participated voluntarily in the study. The participants had at least a 1‐year personal experience of infertility. The data was analysed using inductive content analysis.

**Results:**

The factors that helped the coping of infertile women were personal resources such as mental well‐being and having good abilities to deal with the issue. Also, a well‐functioning relationship and getting help contributed to coping. Coping was also positively influenced by the ability to adapt to a childless life, which included having comforting thoughts, doing things that brought relief and orientating thinking towards the future.

## 1 INTRODUCTION

Infertility or involuntary childlessness is spoken of if pregnancy has not started in a year, despite regular unprotected sexual intercourse, or if there are repeated miscarriages (Tiitinen, 2020a; World Health Organization, 2020; Zegers‐Hochschild et al., 2017). Childlessness can be either intentional due to conscious choice, or unintentional (Zegers‐Hochschild et al., 2017). Infertility can be due to fertility problems or other physiological causes, but also to sex life problems, lifestyle, life situation (Tiitinen, 2020a; WHO, 2020), sexual orientation (WHO, 2020) and trying to have a child at a later age (Tiitinen, 2020a, b; Tulppala, 2012). The need for individually planned infertility treatment is assessed based on examinations (Tiitinen, 2020a). Options include follicular maturation treatments, surgical treatment and fertility treatments (Tiitinen, 2020b).

Having children is often seen as part of the normal course of life. However, infertility is often seen as a psychosocial crisis with strong emotions for the expectant child (Tulppala, 2012), and feelings often fluctuate between hope and despair (Toivanen et al., 2004). Infertility can be experienced as longing or as a huge loss (Miettinen & Rotkirch, 2008). It can be associated with sadness, anxiety, stress, feelings of inferiority (van Rooij et al., 2009; Tulppala, 2012), failure (Toivanen et al., 2004), guilt (van Rooij et al., 2009; Toivanen et al., 2004) and a fear of being left alone and without a family (Tulppala, 2012). The experience of infertility is very painful and permanent, but if it is treated, mental growth is possible and painful emotions no longer dominate the woman's life (Toivanen et al., 2004). Also, in addition to one's own grief, one may have to support one's own parents' grief about a loss of grandparenthood (Kettula‐Pihlaja et al., 2015).

It is estimated that 48 million couples and 186 million individuals worldwide experience infertility. In national policies, infertility prevention, diagnosis and treatment are often not given high priority, and the availability, access and quality of interventions to combat infertility remain a challenge. However, all people have the right to enjoy the highest attainable standard of physical and mental health, and to determine the number, timing and spacing of children. Therefore, addressing infertility is important to ensure that everyone has the right to find a family (WHO, 2020).

## 2 BACKGROUND

Coping refers to a person's efforts to meet external and internal demands that strain and burden their resources. In these efforts, people use methods such as emotional coping, planned problem‐solving, seeking social support, distancing, self‐control, escape and avoidance, positive reassessment of the situation, or negative confrontation (Lazarus, 2012; Pottinger et al., 2016).

The factors that help coping with infertility have not been studied as such, but previous studies show that life experiences and life situations play a role in how we cope (Tulppala, 2012). In the infertility crisis, the spouse plays an important role (Peters et al., 2011), as well as support drawn from other close ones (Kettula‐Pihlaja et al., 2015), peers and professionals (Tulppala, 2012). Openly discussing issues with an understanding and trustworthy person facilitates the handling of emotions (Toivanen et al., 2004), and great comfort and help has been drawn from infertility support groups on the Internet (Malik & Coulson, 2010). Infertility examinations and treatments can contribute to coping (Miettinen & Rotkirch, 2008), although fertility treatments are known to be physically demanding and mentally stressful for a woman. Adapting to the situation has been found to be an important process for coping with infertility (Tulppala, 2012). As a result, the acceptance of different family models may gradually become possible, and the adoption of a child can also be seen as an alternative (Kettula‐Pihlaja et al., 2015).

Because infertility is relatively common and coping with it is important for the individual, the relationship, the family and society, it is therefore necessary and justified to study women's own experiences of the factors that help them with coping.

### 2.1 Research question

This study poses the research question: What are the factors that help the coping of infertile women?

## 3 THE STUDY

### 3.1 Design

This study is qualitative, with the purpose of describing the factors that help the coping of infertile women. The target population of the study was women who had at least a 1‐year personal experience of infertility, and primary infertility still was an on‐going issue for them. The qualitative method was used to specifically explore the experiences of infertile women (Holloway & Galvin, 2016).

### 3.2 Method

Purposive sampling was used in the study. The study was announced by the Finnish Infertility Association *Simpukka ry* in closed Finnish‐language discussion forums on Facebook. An electronic questionnaire with a cover letter was placed on the forum. In this way, participants with the best possible knowledge of the research topic were reached (Holloway & Galvin, 2016). The cover letter gave information on the study, its confidentiality and the voluntary nature of participation (Holloway & Galvin, 2016). The exclusion criteria were: not a woman, <1 year personal experience of infertility, infertility is no longer a topical issue and have biological children.

The research data was gathered 2/2015–1/2017 using an electronic questionnaire which included structured background variable questions and a qualitative open‐ended question related to the factors that help coping. The advantage of open‐ended questions is that they don't lead to a stock response, and instead require the participants to elaborate in their own words, which in turn helps to bring out as many different aspects of the phenomenon as possible (Holloway & Galvin, 2016). In total, 101 aged 22–52 volunteer women who met the criteria of the study target group participated in the study (mean 33.1 years). Almost everyone lived in a relationship that had lasted from 4 months to almost 25 years (mean 8.6 years). The women had been trying to have a child for 1 to 20 years (mean 4.3).

Gathering background information from the participants allowed excluding the non‐target group. Because the research material was collected using an electronic questionnaire, it was not possible to ask follow‐up questions to get more exact answers from the participants. However, the open‐ended question that was used was formally compatible with the purpose of the study and it was answered concisely (Elo et al., 2014).

### 3.3 Analysis

The research data was analysed using inductive content analysis (Elo & Kyngäs, 2008), which made it possible to describe and define the phenomenon under study in a systematic and objectively condensed form. The analytic aim was that the concepts formed from the data reliably describe the phenomenon under study. Original expressions corresponding to the research question (*n* = 635) were extracted from the material, which were then reduced while preserving the essential content. Next, similar expressions were grouped into categories, which were named based on the data. When abstracting the material, subcategories containing similarities were grouped into upper and main categories, and named according to their content. This process formed a conceptual description of the topic under study (Elo & Kyngäs, 2008).

In the responses, the participants shared their own views about the topic that the research question seeks answers to. The original material was reviewed several times during the analysis to ensure the correct interpretation (Elo et al., 2014).

### 3.4 Ethics

The research followed good scientific practice and the ethically responsible and correct practices laid out by the Finnish National Board on Research Integrity (TENK, 2019). Due to the sensitivity of the research topic, the contact information of the project manager was included in the cover letter for possible contact. Permission to conduct the research was requested from *Simpukka ry* and the administrators of the discussion forums. Responding to the questionnaire was interpreted as consent to participate in the study (Iphofen, 2005). Ethical approval was not required.

## 4 RESULTS

The factors that helped the coping of infertile women were personal resources, a well‐functioning relationship, getting help and the ability to adapt to a childless life (Table 1).

**TABLE 1:** The factors that help the coping of infertile women

Main category	Upper category	Subcategory
Personal resources	Mental well‐being	A good mental condition
		Enjoying everyday life
		Maintaining daily routines
		A healthy lifestyle
		Maintaining hope
		The tactful behaviour of others
		The natural attitude of others
	Good abilities to deal with infertility	An outward orientation
		A positive attitude towards life
		A fighting attitude
		Accepting negative emotions
		Emotion control
		Relating infertility to other crises
		Relying on religion
		Using infertility information
A well‐functioning relationship	A good relationship	Satisfaction with a spouse
		Experiencing intimacy
		Being together with a spouse
		Believing in the sustainability of a relationship
	Understanding infertility as a common issue between couples	Sharing an infertility experience with a spouse
		Processing infertility together with a spouse
Getting help	Getting help from the inner circle	The existence of close ones
		Getting support from close ones
		Getting encouragement from close ones
		Becoming understood by close ones
		Getting support from the workplace
		Getting power from a pet
	Obtaining peer support	An awareness of the existence of peers
		Getting peer support from the inner circle
		Getting peer support from different data sources
		Getting peer support from different communities
		Sharing experiences with peers
		Feeling a cohesion with peers
	Infertility treatments	Receiving infertility treatments
		The smooth progression of infertility treatments in the private sector
		Promoting infertility treatments through their own activities
	The feeling of being noticed during infertility treatments	Getting necessary help from nursing staff
		The caring attitude of nursing staff
		Independent decision‐making related to treatments
		Talking to a professional
The ability to adapt to a childless life	Having comforting thoughts	Believing in the expediency of infertility
		Facing the uncontrollability of infertility
		Seeing the benefits of infertility
		Enjoying the benefits of infertility
		Appreciating other important things in life
		Rejoicing in other things
	Doing things that bring relief	Avoiding families with children
		Avoiding the issue of infertility
		Redirecting thoughts to other things
		Spending time with children
		Telling others openly about infertility
		Giving time to oneself
		Dealing with infertility in one's own mind
		Giving help to others
	Orientating thinking towards the future	Accepting infertility as part of one's own life
		Restructuring the future
		Making big life changes

### 4.1 Personal resources

Personal resources to promote coping included mental well‐being and having good abilities to deal with infertility.

#### 4.1.1 Mental well‐being

The mental well‐being that promoted coping was associated with having a good mental condition, enjoying everyday life, maintaining daily routines, having a healthy lifestyle and maintaining hope. In addition, the tactful behaviour of others and their natural attitude contributed to the mental well‐being of infertile women.

Having a good mental condition was described by feelings of life satisfaction, taking care of one's own well‐being, keeping thoughts clear, having good self‐esteem, and listening to and respecting one's own body. Good mental condition was maintained by seeking out things that make one's feel good, pampering oneself, being in good company, managing tasks and activities, and relieving pressure by exercising. At times, maintaining a good mental condition required taking a break from trying to have a child, taking a break in life routines, taking sick leave, or taking sedatives.

The hardest and most important thing has been to stop everything when you can't, which means that being on sick leave has been helpful, although the threshold for taking it was high. (29‐year‐old woman)

Enjoying everyday life included experiencing a good and functional everyday life, enjoying daily routines and doing enjoyable work. In addition, invigorating everyday life with nice things, enjoying culture, hobbies, and nature, doing things by hand, and invigorating oneself while traveling were related to enjoying an everyday life that promotes coping.

Maintaining daily routines involved keeping a regular rhythm of life and the daily rhythm through work and pets. Having a healthy lifestyle involved following a healthy lifestyle and diet, ensuring an adequate amount of rest, maintaining a regular sleep rhythm, exercising and going out.

Maintaining hope included keeping up the hope of having a child (especially because of young age), believing in having a child and also seeing a new opportunity to have a child after a divorce. Hope was also maintained by starting a new treatment, believing in the success of the treatment, reading about the successful infertility treatments of others, gaining hope from the stories of peers and making space for the desired baby at home.

Relating to the tactful behaviour of others, issues that promoted coping were associated with other people not asking about infertility or pushing them to having a child. Taking infertility into account in social interactions also contributed to coping. In addition, the openness of close ones about their own pregnancies, and giving value to a childless family were perceived as the natural attitude of others.

#### 4.1.2 Good abilities to deal with infertility

An outward orientation, a positive attitude towards life, a fighting attitude, accepting negative emotions and having emotional control were perceived as good abilities to deal with infertility. In addition, relating infertility to other crises, relying on religion and using infertility information were related to good abilities to deal with infertility.

Adopting an outward orientation involved having an open, social and talkative nature. A positive attitude towards life included thinking positively, believing in good things, seeing the good sides of a situation and trusting oneself to survive in life. Feeling love, enjoying small moments, maturing with age, cultivating humour and experiencing the irony of life were also included in having a positive attitude to life that promoted coping.

I've been trying to think positively, and that our lives can be good without children, too. (31‐year‐old woman)

The fighting attitude that promoted coping involved women's own perseverance, the ability to gather themselves and gather strength after disappointment, trying to control the situation and focusing on the moment. It also included trying to cope with infertility and making your attitude to life more positive. Accepting negative emotions was also a stance that promoted coping, and included giving way to all kinds of emotions such as grief, anger and rage, as well as going through a range of other emotions. In addition, expressing feelings of fear and despair through writing promoted coping.

It helps when you give yourself permission to feel right. When you get angry, you get rage, when you have grief, you cry. (33‐year‐old woman)

Emotional control involved the conscious exclusion of long‐lasting self‐loathing, grief, bad feelings and anger, as well as stopping keeping up an annoyance of the past and grieving about things to come. It also involved declaring jealousy as useless, and ending the avoidance of families with children. Relating infertility to other crises to promote coping included experiencing earlier life crises and losses, and living with other sorrows. Relying on religion included a reliance on the Christian faith and having a belief in God.

Using infertility information included searching for information on infertility, and especially reading relevant information retrieved from various sources, such as scientific studies. In addition, watching infertility programs and chatting with friends to broaden one's own perspectives contributed to coping.

Reading infertility treatments and related research information from various publications has helped me – the information brings me peace. (28‐year‐old woman)

### 4.2 A well‐functioning relationship

Having a well‐functioning relationship that promotes coping included perceiving a good relationship and understanding infertility as a common issue between couples (Table 1).

#### 4.2.1 A good relationship

Women felt satisfaction with a spouse, experiencing intimacy, being together and believing in the sustainability of a relationship as a good relationship that promoted coping.

Satisfaction with a spouse included experiencing a good and functional relationship, feeling good about everyday life with the spouse, enjoying being together and experiencing a soul partnership. Loving a spouse and feeling happy, as well as sharing common interests with the spouse, also helped coping.

Experiencing intimacy that promoted coping was perceived as intimacy in the relationship, the presence of the spouse and having sex. Being together with a spouse involved doing daily chores, doing hobbies together and traveling. It also included nurturing the romantic side of a relationship, and celebrating dating evenings.

The most important thing of all is to think about some hobby that unites the spouses, otherwise there is a high risk of separation and a vacuum fills the relationship and breaks it (like a new relationship). (50‐year‐old woman)

Believing in the sustainability of a relationship included experiencing the relationship as strong, and trusting its permanence. It also included committing to a spouse, sharing a home with the spouse, becoming accepted by the spouse, continuing the relationship and strengthening the relationship through the experience of infertility.

#### 4.2.2 Understanding infertility as a common issue between couples

The perception of infertility as a common issue involved sharing an infertility experience and processing infertility together with a spouse.

Sharing an infertility experience involved experiencing infertility as a joint project, being with a spouse throughout the infertility process, gaining understanding and comfort, and sharing emotions and crying together. Processing infertility together with a spouse involved unravelling the thoughts about infertility together, discussing things openly, using humour in difficult situations, fostering a spirit of togetherness and fighting on the same side.

I see very clearly the point from which we could have started to perhaps grow apart so that our covenant would not have endured this. But we didn’t do that, we’ve been through these experiences together. (44‐year‐old woman)

### 4.3 Getting help

The women experienced that getting help from their inner circle, obtaining peer support, infertility treatments, as well as the feeling of being noticed during the treatments, had a positive correlation with coping with infertility (Table 1).

#### 4.3.1 Getting help from the inner circle

Women felt the help of the inner circle was derived from the existence of close ones, getting support and encouragement from close ones, and becoming understood by close ones. In addition, coping was facilitated by getting support from the workplace and also power from having a pet.

The existence of close ones involved having a good support network, a trusted family and relatives, and having a wide circle of trusted friends.

Dad and sister also help somewhat just by being there. It becomes apparent that there are even some reasons to go on with life. (34‐year‐old woman)

Getting support from close ones included the involvement of close ones in the treatment process, getting support from close ones who knew about infertility, love between family and friends, and openly discussing infertility with close ones. Getting encouragement from close ones to promote coping involved dealing with infertility with a friend through humour, and doing things together with friends.

My best friend acts as my ‘therapist’ – I deal with it with her through humor. It feels good to joke about this thing at times too, so that it doesn't become a monster inside me. (28‐year‐old woman)

Becoming understood by close ones to promote coping included gaining a hearing by close ones, the understanding attitude of close ones towards infertility, and gaining empathy from close ones. Getting support from the workplace included receiving support from co‐workers, discussing openly about infertility with co‐workers, and receiving support and understanding from a supervisor during infertility treatments. Getting power from a pet involved acquiring a pet and experiencing the pet as a family member. Transferring feelings of tenderness to the pet and satisfying the desire to care with the help of a pet also helped coping.

#### 4.3.2 Obtaining peer support

Women experienced an awareness of the existence of peers, getting peer support from their inner circle, from different data sources and different communities, as well as sharing experiences and feeling a cohesion with peers, as obtaining peer support for coping.

An awareness of the existence of peers that promoted coping was associated with the awareness that infertility also affects others, the prevalence of infertility, hearing about the infertility of others, finding peers and the availability of peer support.

Coping with infertility has been helped by the knowledge that there are other infertile people or people who have lost a child who have experienced that what I am experiencing now. (34‐year‐old woman)

Getting peer support from their inner circle involved an awareness of infertility problems in close ones and about the perspectives of other single relatives, receiving peer support from friends and receiving support from friends who had experienced infertility treatments. Getting peer support from different data sources included peer support from the Internet, literature and movies. Getting peer support from different communities involved receiving support from social media, such as Internet discussion forums and blogs, peer support groups for infertile people and joining an infertility association. Sharing experiences with peers included talking to peers and talking about feelings about infertility, telling peers about unpleasant thoughts of bitterness and experiences of injustice, and writing to peers about things that make them feel bad. Feeling a cohesion with peers included gaining understanding and compassion from peers, and experiencing being on the same side as peers.

Peer support has become very important, because even though there are listeners around me anyway (and we've already told to our inner circle about our infertility quite openly now), I don't have people who understand me near me. (30‐year‐old woman)

#### 4.3.3 Infertility treatments

Women experienced coping‐promoting via infertility treatments in regard to receiving infertility treatments, the smooth progression of infertility treatments in the private sector and promoting infertility treatments through their own activities.

Receiving infertility treatments involved gaining access to infertility treatments, quick access to treatments and the progress of treatments. The smooth progression of infertility treatments in the private sector included switching from public health care to a private infertility clinic, and on the private side, attending infertility treatments, as well as the way that things are dealt with in the sector. Promoting infertility treatments through their own activities included influencing the progress of infertility treatments, preparing one's own body for future treatments, trying out natural products and natural treatments, and doing everything possible in relation to the treatments.

#### 4.3.4 The feeling of being noticed during infertility treatments

The women felt that getting necessary help from nursing staff, their caring attitude, having independent decision‐making related to treatment and talking to a professional as creating the feeling of being noticed during infertility treatments.

Getting necessary help from nursing staff to promote coping was accompanied by getting equal information on infertility treatments in health care, and receiving encouragement and support from nursing staff. It also involved feeling that the nursing staff handle their affairs well, they take care of them and trust in the success of the treatment, as well as focusing on controlling possible diseases and taking good care of any pain when the treatments have finished. In addition, obtaining necessary assistance involved obtaining a doctor's recommendation to stop treatment, and support with a child adoption decision.

… I think it would be good for each couple to be told the possibilities, the probabilities, and in addition, what research opportunities can offer and where the opportunities for public‐sector care run out. (30‐year‐old woman)

The caring attitude of nursing staff was associated with experiencing the nursing staff as pleasant, gaining their empathy and individual attention, and their having a genuine interest towards the couple.

Independent decision‐making related to treatments included taking a break from infertility treatments, making independent decisions about continuing treatments and considering alternatives such as experimenting with gift cells, in peace. Talking to a professional such as a psychiatric nurse or psychologist, and attending psychotherapy was perceived as getting help that supported coping.

### 4.4 The ability to adapt to a childless life

Coping was positively influenced by the ability to adapt to a childless life, which included having comforting thoughts, doing things that bring relief and orientating thinking towards the future (Table 1).

#### 4.4.1 Having comforting thoughts

Women experienced having comforting thoughts as believing in the expediency of infertility, facing the uncontrollability of infertility, seeing and enjoying the benefits of infertility, appreciating other important things in life and rejoicing in other things.

Believing in the expediency of infertility that promotes coping involved perceiving infertility as a matter of fate and part of the life cycle, and believing in the appropriateness of events and being childless as another kind of life direction.

I now believe that before we were born, we have (or have chosen) guidelines for our lives. Infertility is "my destiny," without that experience I wouldn't be the person I am right now. (50‐year‐old woman)

Facing the uncontrollability of infertility involved finding the cause of infertility, while on the other hand, remaining unclear and understanding the uncontrollability of infertility. Seeing the benefits of infertility included appreciating the ease of doing things compared to families with children, sharing love with a spouse between two and thinking about the world as being overpopulated.

Enjoying the benefits of infertility that promoted coping included enjoying a long night's sleep, experiencing the freedom of doing things, planning new hobbies, and leaving time and energy to make other dreams come true. Appreciating other important things in life included experiencing life meaningfully without a child, and thirsting for information about life and the world. Rejoicing in other things was reflected in an appreciation of the many joys of life, and things that create a good mood.

#### 4.4.2 Doing things that bring relief

Women felt that avoiding families with children and the issue of infertility, redirecting their thoughts to other things, and spending time with children, were things that brought relief to promote coping. In addition, they experienced that telling others openly about infertility, giving time to oneself, dealing with infertility in one's own mind and giving help to others were also beneficial.

Avoiding families with children involved avoiding pregnant women and babies, and having limited contact with families with children. Avoiding the issue of infertility included avoiding related discussion forums and reading infertility blogs, avoiding those who are curious about having a child, and disconnecting with other infertile people. Redirecting thoughts to other things included placing infertility out of mind, for example, by keeping oneself busy, and focusing on things other than infertility treatments, trying to have a child, and a wish to get pregnant. In addition, it also included immersing oneself in work and study, directing thoughts to other things such as acting as a supporting family, and thinking about the journey ahead on bad days.

… I'm currently doing my second master's degree and doing my master's thesis has been an escape from the gruesome reality of being childless. (28‐year‐old woman)

Spending time with children to promote coping included serving as a supportive family, the existence of surrogate children, being close to children and godchildren, feeling like the children of siblings were one's own and taking care of them, and having an adopted child and rejoicing in it. Telling others openly about infertility openly involved talking openly about infertility, saying things out loud, and writing a blog. Giving time to oneself was associated with the passage of time, for example, after failed treatment, going through difficulties over time, and the distribution of infertility‐related process‐like grief work over a long time.

The best medicine is time, doing its job. (26‐year‐old woman)

Dealing with infertility in one's own mind included curling inward, and processing infertility through one's own thoughts, writing, and also through books, music and films about infertility. Giving help to others involved volunteering, listening to other sorrows more sensitively, helping other people's children and supporting others.

#### 4.4.3 Orientating thinking towards the future

Women perceived coping as orientating their thinking towards the future, to accepting infertility as part of one's own life, re‐structuring the future and making big life changes.

Accepting infertility as part of one's own life that promotes coping involved a fading of hope, a belief in one's own ability to accept infertility, and an understanding of the finality of infertility. In addition, it involved understanding the unfairness of life, contenting oneself with the existing reality, the continuation of life and striving for a full life.

I try to think that you can't have everything in life. (40‐year‐old woman)

Restructuring the future involved rethinking the content of life, seeing other goals in addition to motherhood, making alternative plans about the future, trying to enjoy all aspects of life and thinking about adoption. Making big life changes to promote coping involved resigning from work, aiming for a dream job, taking up studies, going to a new school and planning to do a dissertation. In addition, in some cases, it led to divorce, and in one case, the adoption of a daughter.

I saw how I changed in a physically worse direction, I lost 10 kg in a couple of months, I was so deep in my own misery and I thought it didn’t matter anymore. Realizing my own self‐destructive behavior, I began to think about the future and what I could still do in my life. (33‐year‐old woman)

## 5 DISCUSSION

The results of the study show that the personal resources of infertile women and strengthening those resources are important for coping. Mental well‐being and having good abilities to deal with infertility contribute to the coping of women. The study highlighted the importance of having a good mental condition, the enjoyment of everyday life, daily routines and having a healthy lifestyle in coping for women. The research supports the previously found benefits of maintaining hope (Lee et al., 2010; van Rooij et al., 2009) and maintaining a positive attitude to life (Lee et al., 2010; Peters et al., 2011) in coping. An outward orientation and having a fighting attitude emerged as new ways to promote women's coping. In addition, the study confirmed the importance of raising awareness of the difficult emotions related to infertility (Toivanen et al., 2004) and giving space to emotions and their processing (Tulppala, 2012), which is why enabling the discussion of emotions is important in health care.

The study gives a broader picture of the importance of a well‐functioning relationship in the coping of infertile people, which is why it is worth investing in supporting and strengthening the relationship. According to the results, satisfaction with a spouse, experiencing intimacy in a relationship, believing in the sustainability of the relationship, and sharing and processing the experience of infertility together helped women to cope with infertility. The results support previous information on the importance of a spouse (Peters et al., 2011; Tulppala, 2012) and reciprocity of a relationship (Tulppala, 2012) in the infertility crisis. For this reason, it would be important, for example, to offer relationship therapy to infertile couples.

According to the study, the smooth progression of infertility treatments seen in the private sector helps coping, so infertility treatments should be developed, particularly in the public sector. The results emphasized the importance of the feeling of being noticed during infertility treatments, and also the caring attitude of nursing staff. The results confirm that mental support by nurses and processing infertility are important nursing interventions throughout the treatment process (Toivanen et al., 2004). Arranging time to consider the psyche, for example, through conversations with a nurse is important. The study also confirmed the importance of support received from the social environment in coping with infertility (Kettula‐Pihlaja et al., 2015; Sormunen et al., 2018). Therefore, there is a need for different forms of peer support and peer support to be readily available. Peer support groups, arranged by nurses, could also be more broadly targeted for those who no longer have any hope of having a child.

According to this study, supporting women to adapt to a childless life is also important, as has been shown in an earlier study (Tulppala, 2012). The results emphasize finding comforting thoughts so that infertile women can experience the meaning of life, and feel the joy of life. Nurses can help the childless realize their own resources and find their own way to live, which helps them to cope. Research also shows that spending time with children and giving help to others promotes coping. However, other research results are parallel in that social isolation and the avoidance of children (Toivanen et al., 2004) and unpleasant situations (Peterson et al., 2006) such as leaving situations when children are being talked about (Sormunen et al., 2018), and directing thoughts to other matters (van Rooij et al., 2009) can also help with coping. The results confirm that women need time to deal with infertility in peace (Kettula‐Pihlaja et al., 2015; Tulppala, 2012), although an active handling of the issue is seen as a good thing (Lee et al., 2010; Peterson et al., 2006). The results also confirm the importance of a future orientation of thoughts for coping (Kettula‐Pihlaja et al., 2015; Peters et al., 2011). Furthermore, nurses can help infertile women by supporting the re‐structuring of their life content and encouraging them to make big life changes that may help in this context.

### 5.1 Reliability and limitations

The reflexivity of the results was strengthened by working on the phenomenon for a long time and cooperating with the research group at different stages. The progress of the research process is clearly and comprehensibly described to ensure the validity of the research. The progress of the analysis and the process leading to the conclusions of the results are illustrated with initial expressions, which increases the credibility of the study's findings. However, it is acknowledged that recruiting participants from Facebook's closed forums affects the portability of results because site visitors actively seek peer support. Those who reflect on infertility alone may therefore have been left out from the study. In regard to sample size and saturation, the data was collected over 2 years and thus enabled a large number of study participants to be recruited and a saturation of data to be reached. Due to the number of participants in the study and the rich material, it can therefore be assumed that the phenomenon has been described in versatile ways, and that the results have a good degree of transferability, at least in the Finnish context (Cope, 2014; Holloway & Galvin, 2016). However, the replication of the study in other national and international contexts would give a broader picture of the coping mechanisms of infertile women, and the issues involved.

## 6 CONCLUSION

Based on the research results, the following conclusions can be drawn:

- The coping of infertile women can be promoted by supporting and strengthening their personal resources as well as the relationship between couples.
- Coping with infertility largely occurs in a woman's mind at the level of emotions and thoughts, so discussing feelings and thoughts is an important way to support coping.
- Arranging relationship therapy and various forms of peer support for infertile women and couples may help them to cope with infertility.
- During infertility examinations and treatments, women need discreet treatment, factual information and emotional support.

## CONFLICT OF INTEREST

The authors have no conflict of interest to declare.

## AUTHOR CONTRIBUTIONS

The study design: STH, ALA; data collection: ALA; data analysis and interpretation, drafting the manuscript: STH; revising the manuscript critically for important intellectual content, commenting and editing: MK, ALA. All authors have given final approval of the version to be published.

## Data Availability

The data that support the findings of this study are available on request from the corresponding author. The data are not publicly available due to privacy or ethical restrictions.
